# Crystal structure of bis­(μ-4-nitro­benzoato-κ^2^ *O*:*O*′)bis­[bis­(4-methyl­pyridine-κ*N*)(4-nitro­benzoato-κ^2^ *O*,*O*′)manganese(II)]

**DOI:** 10.1107/S2056989016015589

**Published:** 2016-10-11

**Authors:** Sourav J. Bharali, Sanchay J. Bora, Birinchi K. Das

**Affiliations:** a Department of Chemistry, Gauhati University, Guwahati 781 014, India; b Department of Chemistry, Pandu College, Guwahati 781 012, India

**Keywords:** crystal structure, manganese 4-nitro­benzoate, Mn^II^ tetra­carboxyl­ate dimer, asymmetric carboxyl­ate bridge

## Abstract

The title compound, [Mn_2_(C_7_H_4_NO_4_)_4_(C_6_H_7_N)_4_] or [Mn_2_(μ-NBz)_2_(κ^2^-NBz)_2_(4-Mepy)_4_], where NBz is 4-nitro­benzoate and 4-Mepy is 4-methyl­pyridine, is a centrosymmetric dinuclear complex in which the Mn^II^ atoms are bridged by two NBz ligands with Mn⋯Mn = 4.1324 (4) Å. The Mn^II^ atom in this dimeric species is present in a distorted octa­hedral environment with the four coordinating O atoms forming the equatorial plane and the two pyridyl N atoms occupying the axial sites. An important structural feature of the dimeric complex is that each of the bridging carboxyl­ate ligands binds to the metal ions in an asymmetric fashion involving bent and linear Mn—O—C units. The crystal packing is consolidated by C—H⋯O and C—H⋯π interactions.

## Abstract 2

The crystal structure of a dinuclear tetra­carboxyl­ate complex of manganese(II) is reported wherein the Mn^II^ atoms are bridged by two carboxyl­ate anions.

## Chemical context   

Polynuclear manganese complexes with carboxyl­ate ligation have received great attention due to their potential applications in catalysis (Arafa *et al.*, 2014 ▸), magnetism (Miyasaka *et al.*, 2004 ▸) and their anti­tumor activity (Dey *et al.*, 2015 ▸) as well as in other areas. The occurrence of Mn in a number of oxidation states (II–IV) under normal conditions and also the ability of carboxyl­ato ligands to display a variety of coordin­ation modes are the main reasons why Mn–carboxyl­ates have received a lot of attention in the recent past. It has been reported that an Mn-based binuclear complex of composition [Mn_2_(bbppnol)(μ-O_2_CCH_3_)_2_] [bbppnol = *N*,*N*′-bis­(2-hydroxy­benz­yl)*N*,*N*′-bis­(2-methyl­pyrid­yl)-2-ol-1,3-propanediamine] with two bridging acetato ligands is active as a catalyst in the epoxidation of cyclo­hexene and cyclo­octene (Castaman *et al.*, 2009 ▸). A series of dimeric complexes with the general formula [Mn_2_(O_2_CCH_3_)*L*] {where *L* = 2,2′-[2-hy­droxy-5-(pivalamido­meth­yl)-1,3-phenyl­ene]bis­(1*H*-benzo[*d*]imidazole-4-carb­oxy­lic acid), 2,2′-(5-benzyl-2-hy­droxy-1,3-phenyl­ene)bis­(1*H*-benzo[*d*]imidazole-4-carb­oxy­lic acid) *etc*.} have been explored as catalysts for the water-oxidation reaction with a view to generating O_2_ and H_2_ (Arafa *et al.*, 2014 ▸). Microwave-assisted alcohol oxidation with *tert*-butyl­hydro­peroxide (TBHP) has been carried out (Sutradhar *et al.*, 2014 ▸) using a Schiff base-containing Mn dimer. Manganese complexes are also recognized for their magnetic behaviour since coordination compounds of this metal often display large ground-state spin (*S*) values and the polynuclear manganese cluster [Mn_12_O_12_(CH_3_COO)_16_(H_2_O)_4_]·2CH_3_COOH·4H_2_O is considered to be the first single mol­ecule magnet (SMM) (Uhrecký *et al.*, 2013 ▸; Sessoli *et al.*, 1993 ▸). Complexes of manganese are also considered to be important in view of the occurrence of an Mn_4_Ca unit in the active site of Photosystem II that catalyses the water-splitting reaction to evolve oxygen in nature (Nocera, 2012 ▸).
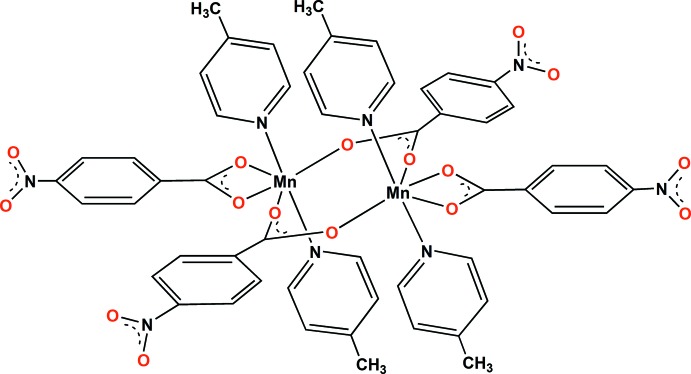


Keeping in mind earlier results published from our laboratory (Chakrabarty *et al.*, 2007 ▸) on the synthesis and catalytic properties of cobalt(III)–oxide pseudo-cubane units of the type [Co_4_O_4_(μ-O_2_C*R*)_4_ *L*_4_], where *R* is an alkyl or aryl group and *L* is a monodentate pyridyl ligand, and also due to their relevance as catalysts for the water-oxidation reaction (McCool *et al.*, 2011 ▸), we explored whether analogous manganese complexes could also be synthesized. These efforts have led to the synthesis of the title complex, among others. Herein we report the synthesis, crystal structure and some salient properties of the dimeric manganese(II) compound [Mn_2_(μ-NBz)_2_(κ^2^-NBz)_2_(4-Mepy)_4_], **I** [Chem scheme1], which belongs to a structure type constituted of only a limited number of complexes (*vide infra*).

## Structural commentary   

**Figure 1:**
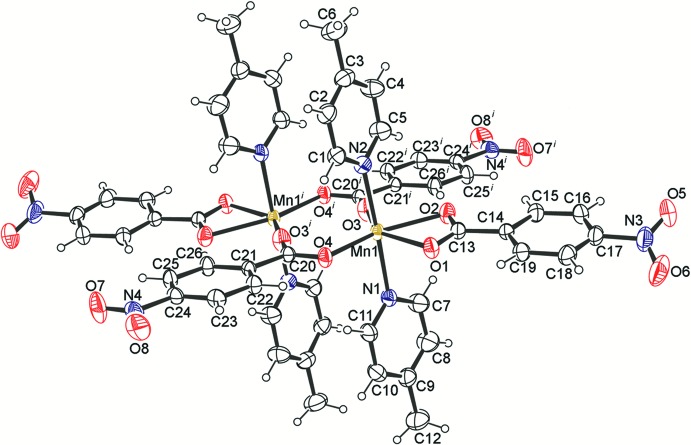
An *ORTEP*-style view of the mol­ecular structure of [Mn_2_(μ-NBz)_2_(κ^2^-NBz)_2_(4-Mepy)_4_] **I** [Chem scheme1] with displacement ellipsoids drawn at the 50% probability level.

Fig. 1 ▸ shows the mol­ecular structure of the dimeric complex. The two Mn atoms are related by an inversion centre and are bridged by the carboxyl­ate anions of two NBz ligands in a *syn–syn* fashion. Each Mn^II^ atom is further coordinated by a carboxyl­ato ligand in chelating mode. The four oxygen atoms – two from a pair of bridging NBz ligands and two from a chelating NBz ligand - are nearly coplanar with each of the central Mn atoms, forming an equatorial plane; the axial positions for both are occupied by two 4-methyl­pyridine ligands completing the distorted octa­hedral geometry around each Mn^II^ atom. The bridging Mn—O(carbox­yl) bond lengths (∼2.1 Å) are found to be shorter than the Mn—O(carbox­yl) distances (∼2.3 Å) in the chelating ligands (Table 1 ▸). For the chelating NBz anions, the longer Mn—O distances can be attributed to the steric crowding imposed by the neighbouring bridging bis-monodentate NBz anions.

The Mn⋯Mn distance of 4.1324 (4) Å in **I** [Chem scheme1] precludes any direct bonding inter­action between the Mn^II^ atoms and is comparable to the corresponding distances in the structurally related Co^II^ complexes [{Co(dpe)(NO_2_BDC)}·0.5(dpe)]_*n*_·*n*H_2_O (4.181 Å; Luo *et al.* 2003 ▸), [Co_2_(4,4′-bipy)_2_(O_2_CC_6_H_5_)_4_]_*n*_ (4.060 Å; Zhang *et al.* 2007 ▸) and Co_2_(μ-4-nbz)_2_(κ^2^-4-nbz)_2_(4-CNpy)_4_ (4.226 Å; Chakravorty & Das, 2016 ▸). However, it is considerably shorter than in its most closely related analogue [Mn_2_(μ-OBz)_2_(κ^2^-OBz)_2_(py)_4_] in which the Mn⋯Mn separation is 4.531 Å (Ran *et al.*, 2006 ▸).

The highly distorted nature of the MnO_4_N_2_ octa­hedron in the title species, which is probably due to the steric crowding of both the bridging and chelating NBz ligands surrounding the Mn^II^ atom, is manifested by the O—Mn—O and O—Mn—N angles. While the former are in the range 56.95 (4)–150.77 (4)°, the latter are in the range 88.02 (5)–94.59 (5)°.

In the title compound, the carboxyl –COO and –NOO planes of the chelating NBz anion deviate slightly from the phenyl ring plane, forming dihedral angles of 2.6 (3) and 23.6 (4)°, respectively. According to Kaduk (2000 ▸) and Kaduk & Golab (1999 ▸), completely planar phenyl carboxyl­ates are associated with low conformational energy and any deviation from planarity leads to an increase in the energy of the system. However, this destabilization can be compensated for by efficient crystal packing in the solid state.

## Supra­molecular features   

**Figure 2:**
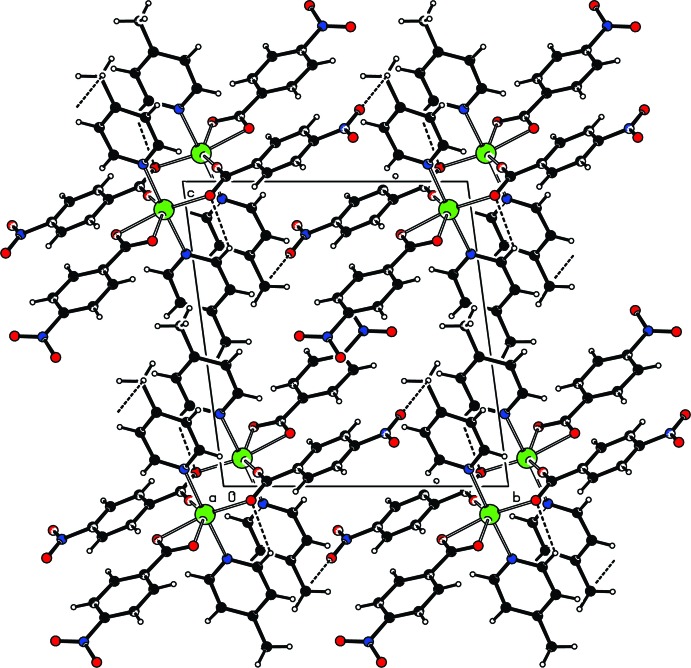
Packing diagram showing C—H⋯O inter­actions (dashed lines) in the crystal structure of **I** [Chem scheme1].

**Figure 3:**
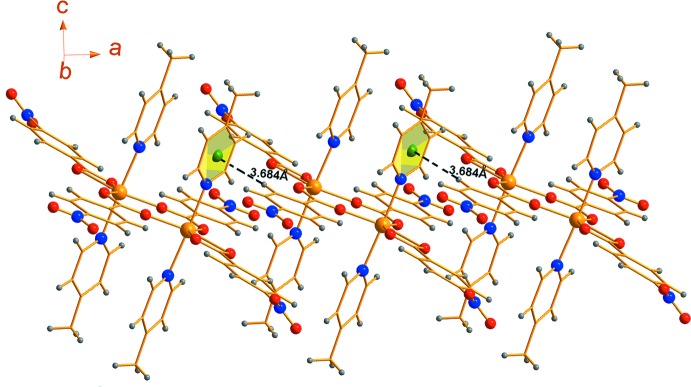
Inter­molecular C—H⋯π inter­actions observed between phenyl-ring H atoms of NBz and phenyl ring π-systems of 4-Mepy in the crystal structure of **I** [Chem scheme1].

The crystal structure of **I** [Chem scheme1] features several intra­molecular as well as inter­molecular C—H⋯O inter­actions wherein the O atoms from –NO_2_ and –CO_2_ groups of the NBz ligand act as hydrogen acceptors (Table 2 ▸ and Fig. 2 ▸). While the *D*⋯*A* separations for these weak contacts are in the range of 3.161 (2) to 3.369 (2) Å, the <C—H⋯O angles are generally lower than 130°, except in one case where a hydrogen bond with a greater *D*⋯*A* separation of 3.369 (2) Å forms has an angle of 172°. In addition, inter­molecular C—H⋯π inter­actions involving the pyridyl ring π system of the 4-Mepy ligand link the complex mol­ecules into chains along the *a* axis (Fig. 3 ▸). Although each of the above non-covalent contacts is individually weak, the presence of many of these supra­molecular contacts clearly result in extra stability of the species in the solid state. Indeed, the involvement of the –NO_2_ and –CH_3_ groups at the 4-positions of the phenyl ring of the NBz ligand and the pyridyl ring of the 4-Mepy ligand may explain why the isolation of complexes analogous to **I** [Chem scheme1] has not been possible for some combinations of carboxyl­ato and pyridyl ligands.

## Database survey   

A survey of the Cambridge Structural Database (Groom *et al.* 2016 ▸) shows that only a few dinuclear Mn complexes with both bridging and chelating carboxyl­ate linkages are known. We have tabulated some of the available data for complexes of the type [Mn_2_(μ-O_2_CR)_2_(κ^2^-O_2_CR)_2_ *L*_4_] in Table 3 ▸ in order to compare some of the important geometric parameters. For all complexes, the Mn—O bonds involving the chelating carboxyl­ato ligands are longer than the corresponding Mn—O bonds in the bridging carboxyl­ato ligands. Of particular note among the listed parameters is the near linearity of one of the the <Mn—O—C angles [178.20 (1)° and 116.68 (8)°] observed in the crystal structure of **I** [Chem scheme1]. For its most closely related known species, [Mn_2_(μ-OBz)_2_(κ^2^-OBz)_2_(py)_4_] (Ran *et al.* 2006 ▸), the corresponding angles are 149.32 (1) and 133.39 (1)°, respectively. The more pronounced asymmetry of bonding in the bridging carboxyl­ato groups in **I** [Chem scheme1] may be ascribed to steric factors and also to differences in mol­ecular packing effects resulting from the presence of substituents on the aromatic rings of both types of ligand.

## Synthesis and crystallization   

A mixture of MnSO_4_·H_2_O (0.845 g, 5 mmol), NaNBz (1.89 g, 10 mmol) and 4-Mepy (1 ml, 10 mmol) was stirred mechanically in water (20 ml) at room temperature for 4 h. The yellow precipitate that appeared was washed thoroughly with water and then with methanol before being dried in a vacuum desiccator over fused CaCl_2_. Yield: 2.58 g (85% based on Mn). Light-yellow transparent crystals of **I** [Chem scheme1] suitable for X-ray analysis were obtained in 2–3 days from a solution prepared by mixing 2 ml of a methano­lic solution of NaNBz (1 mmol) with a solution (2 ml) of MnSO_4_·H_2_O (0.5 mmol) containing 4-Mepy (1 mmol) in methanol/water (1:1 *v*/*v*). Analysis calculated for C_48_H_36_N_8_O_16_Mn_2_: C, 52.84%; H, 3.30%; N, 10.27%; found: C, 52.04%; H, 3.02%; N, 9.8%; μ_eff_ (295 K)/Mn = 5.36 BM.

The method developed by us to prepare **I** [Chem scheme1] is simpler than the reported procedure for preparing the related species [Mn_2_(μ-OBz)_2_(κ^2^-OBz)_2_(py)_4_] (Ran *et al.* 2006 ▸) and the present method can be easily extended to obtain other analogous manganese(II) complexes.

## Refinement   

Crystal data, data collection and structure refinement details are summarized in Table 4 ▸. Hydrogen atoms were positioned geometrically (aromatic C—H = 0.93 Å, methyl C—H = 0.96 Å) and were included in the refinement in the riding-model approximation, with *U*_iso_(H) set at 1.2–1.5*U*_eq_(C).

## Supplementary Material

Crystal structure: contains datablock(s) I. DOI: 10.1107/S2056989016015589/vn2116sup1.cif

Structure factors: contains datablock(s) I. DOI: 10.1107/S2056989016015589/vn2116Isup2.hkl

CCDC reference: 1508004

Additional supporting information: crystallographic information; 3D view; checkCIF report

## Figures and Tables

**Table 1:** Selected geometric parameters (Å, °)

Mn1—O3	2.1122 (10)	O1—C13	1.2460 (17)
Mn1—O4	2.1328 (9)	O3—C20^i^	1.2358 (15)
Mn1—N2	2.2621 (12)	O4—C20	1.2523 (16)
Mn1—O2	2.2672 (11)	O7—N4	1.230 (3)
Mn1—N1	2.2746 (13)	N4—O8	1.198 (3)
Mn1—O1	2.3285 (11)	N3—O6	1.204 (2)
O2—C13	1.2495 (17)	N3—O5	1.212 (2)
			
O3—Mn1—O4	120.82 (4)	O3—Mn1—O1	144.94 (4)
O3—Mn1—N2	89.91 (5)	O4—Mn1—O1	94.05 (4)
O4—Mn1—N2	89.04 (4)	N2—Mn1—O1	94.59 (4)
O3—Mn1—O2	88.38 (4)	O2—Mn1—O1	56.95 (4)
O4—Mn1—O2	150.77 (4)	N1—Mn1—O1	88.02 (5)
N2—Mn1—O2	89.75 (5)	C13—O2—Mn1	91.41 (8)
O3—Mn1—N1	88.27 (5)	C13—O1—Mn1	88.68 (8)
O4—Mn1—N1	90.22 (4)	C20^i^—O3—Mn1	178.20 (10)
N2—Mn1—N1	177.33 (4)	C20—O4—Mn1	116.68 (8)
O2—Mn1—N1	92.15 (4)		

Symmetry code: (i) 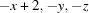.

**Table 2:** Hydrogen-bond geometry (Å, °) *Cg* is the centroid of the N2/C1–C5 ring.

*D*—H⋯*A*	*D*—H	H⋯*A*	*D*⋯*A*	*D*—H⋯*A*
C1—H1⋯O4	0.93	2.61	3.172 (2)	119
C2—H2⋯O1^ii^	0.93	2.65	3.278 (2)	125
C11—H10⋯O4	0.93	2.55	3.161 (2)	124
C22—H25⋯*Cg* ^ii^	0.93	2.80	3.6844 (16)	160

Symmetry code: (ii) 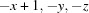.

**Table 3:** Comparison of geometrical parameters (Å, °) for [Mn_2_(μ-NBz)_2_(κ^2^-NBz)_2_(4-Mepy)_4_] **I** [Chem scheme1] and structurally related Mn^II^–carboxyl­ate complexes

Compound	Mn⋯Mn	Mn—O—C	*M*—O (chelate)	*M*—O(bridge)
[Mn_2_(μ-NBz)_2_(κ^2^-NBz)_2_(4-Mepy)_4_]^*a*^	4.1324 (4)	178.20 (1), 116.68 (8)	2.267 (1), 2.329 (1)	2.112 (1), 2.132 (1)
[Mn_2_(μ-tolf)_2_(κ^2^-tolf)_2_(bipyam)_2_]^*b*^	4.548	150.37 (2), 139.28 (2)	2.215 (2), 2.363 (2)	2.087 (2), 2.102 (2)
[Mn_2_(μ-OAc)_2_(κ^2^-OAc)_2_(L1)_2_]^*c*^	4.160	151.50 (3), 127.72 (3)	2.280 (3), 2.294 (3)	2.142 (5), 2.280 (4)
[Mn_2_(μ-OBz)_2_(κ^2^-OBz)_2_(py)_4_]^*d*^	4.531	149.32 (1), 133.39 (1)	2.305 (1), 2.232 (1)	2.109 (1), 2.094 (1)
[Mn_2_(μ-DFBz)_2_(κ^2^-DFBz)_2_(THF)_2_]^*e*^	4.299	155.76 (3), 131.40 (3)	2.194 (3), 2.226 (3)	2.061 (4), 2.040 (3)

Notes: (*a*) present work (HNBz is 4-nitro­benzoic acid and 4-Mepy is 4-methyl­pyridine); (*b*) Zampakou *et al.* (2014 ▸) (Htolf is tolfenamic acid and bipyam is 2,2′-bi­pyridyl­amine); (*c*) Mukherjee *et al.* (2004 ▸) (HOAc is acetic acid and *L*1 is 1,8-bis­(4-pyridyl­ethyn­yl)anthracene); (*d*) Ran *et al.* (2006 ▸) (HOBz is benzoic acid); (*e*) Sivanesan *et al.* (2014 ▸) (HDFBz is 2,6-di(4-fluoro­phen­yl)benzoic acid).

**Table 4:** Experimental details

Crystal data	
Chemical formula	[Mn_2_(C_7_H_4_NO_4_)_4_(C_6_H_7_N)_4_]
*M*_r_	1146.83
Crystal system, space group	Triclinic, *P* 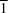
Temperature (K)	293
*a*, *b*, *c* (Å)	8.8337 (3), 12.4240 (4), 12.9995 (4)
α, β, γ (°)	94.357 (1), 99.607 (1), 107.270 (1)
*V* (Å^3^)	1331.28 (7)
*Z*	1
Radiation type	Mo *K*α
μ (mm^−1^)	0.55
Crystal size (mm)	0.28 × 0.24 × 0.18

Data collection	
Diffractometer	Bruker SMART APEXII CCD
Absorption correction	Multi-scan (*SADABS*; Bruker, 2012 ▸)
No. of measured, independent and observed [*I* > 2σ(*I*)] reflections	31721, 7705, 6595
*R*_int_	0.022
(sin θ/λ)_max_ (Å^−1^)	0.704

Refinement	
*R*[*F* ^2^ > 2σ(*F* ^2^)], *wR*(*F* ^2^), *S*	0.036, 0.110, 1.03
No. of reflections	7705
No. of parameters	354
H-atom treatment	H-atom parameters constrained
Δρ_max_, Δρ_min_ (e Å^−3^)	0.33, −0.25

Computer programs: *APEX2* (Bruker, 2012 ▸), *SAINT* (Bruker, 2012 ▸), *SHELXT2013* (Sheldrick, 2015*a* ▸), *SHELXL2013* (Sheldrick, 2015*b* ▸), *ORTEP-3 for Windows* (Farrugia, 2012 ▸), *WinGX* (Farrugia, 2012 ▸), *PLATON* (Spek, 2009 ▸), *DIAMOND* (Brandenburg, 2006 ▸) and *publCIF* (Westrip, 2010 ▸).

## supplementary crystallographic information

### Crystal data

**Table:**

[Mn_2_(C_7_H_4_NO_4_)_4_(C_6_H_7_N)_4_]	*Z* = 1
*M**_r_* = 1146.83	*F*(000) = 590
Triclinic, *P*1	*D*_x_ = 1.430 Mg m^−^^3^
*a* = 8.8337 (3) Å	Mo *K*α radiation, λ = 0.71073 Å
*b* = 12.4240 (4) Å	Cell parameters from 31721 reflections
*c* = 12.9995 (4) Å	θ = 2.5–30.0°
α = 94.357 (1)°	µ = 0.55 mm^−^^1^
β = 99.607 (1)°	*T* = 293 K
γ = 107.270 (1)°	Prism, yellow
*V* = 1331.28 (7) Å^3^	0.28 × 0.24 × 0.18 mm

### Data collection

**Table:**

Bruker SMART APEXII CCD diffractometer	6595 reflections with *I* > 2σ(*I*)
Radiation source: Sealed X-ray Tube	*R*_int_ = 0.022
phi and ω scans	θ_max_ = 30.0°, θ_min_ = 2.5°
Absorption correction: multi-scan (SADABS; Bruker, 2012)	*h* = −12→12
	*k* = −15→17
31721 measured reflections	*l* = −18→18
7705 independent reflections	

### Refinement

**Table:**

Refinement on *F*^2^	0 restraints
Least-squares matrix: full	Hydrogen site location: inferred from neighbouring sites
*R*[*F*^2^ > 2σ(*F*^2^)] = 0.036	H-atom parameters constrained
*wR*(*F*^2^) = 0.110	*w* = 1/[σ^2^(*F*_o_^2^) + (0.0619*P*)^2^ + 0.2308*P*] where *P* = (*F*_o_^2^ + 2*F*_c_^2^)/3
*S* = 1.03	(Δ/σ)_max_ = 0.001
7705 reflections	Δρ_max_ = 0.33 e Å^−^^3^
354 parameters	Δρ_min_ = −0.25 e Å^−^^3^

### Special details

**Table:**

Geometry. All esds (except the esd in the dihedral angle between two l.s. planes) are estimated using the full covariance matrix. The cell esds are taken into account individually in the estimation of esds in distances, angles and torsion angles; correlations between esds in cell parameters are only used when they are defined by crystal symmetry. An approximate (isotropic) treatment of cell esds is used for estimating esds involving l.s. planes.

### Fractional atomic coordinates and isotropic or equivalent isotropic displacement parameters (Å^2^)

**Table:**

	*x*	*y*	*z*	*U*_iso_*/*U*_eq_	
Mn1	0.88391 (2)	0.07843 (2)	0.09236 (2)	0.03854 (7)	
N1	1.00416 (16)	0.02174 (10)	0.23777 (10)	0.0501 (3)	
N2	0.77004 (14)	0.13048 (10)	−0.05684 (9)	0.0468 (2)	
C1	0.62898 (18)	0.06804 (13)	−0.11848 (12)	0.0520 (3)	
H1	0.5724	−0.0002	−0.0982	0.062*	
C2	0.5640 (2)	0.09997 (16)	−0.21003 (13)	0.0605 (4)	
H2	0.4657	0.0536	−0.2502	0.073*	
C4	0.7884 (2)	0.26701 (18)	−0.17717 (16)	0.0701 (5)	
H4	0.8456	0.3367	−0.1947	0.084*	
C5	0.8460 (2)	0.22967 (16)	−0.08665 (15)	0.0629 (4)	
H5	0.9425	0.2755	−0.0440	0.075*	
C3	0.6444 (2)	0.20080 (17)	−0.24265 (13)	0.0617 (4)	
C11	0.9461 (3)	−0.08110 (14)	0.26426 (14)	0.0697 (5)	
H10	0.8573	−0.1330	0.2190	0.084*	
C7	1.1308 (2)	0.09397 (17)	0.30492 (16)	0.0734 (5)	
H6	1.1740	0.1670	0.2885	0.088*	
C9	1.1406 (3)	−0.04168 (18)	0.42468 (14)	0.0672 (4)	
C8	1.2006 (3)	0.0656 (2)	0.39734 (17)	0.0845 (6)	
H7	1.2888	0.1191	0.4416	0.101*	
C10	1.0100 (3)	−0.11549 (16)	0.35530 (16)	0.0805 (6)	
H9	0.9644	−0.1890	0.3698	0.097*	
O2	0.94565 (14)	0.25815 (9)	0.17435 (9)	0.0561 (3)	
O1	0.72129 (14)	0.13417 (9)	0.19520 (9)	0.0562 (3)	
C13	0.82251 (17)	0.23111 (11)	0.21416 (10)	0.0435 (3)	
C14	0.79795 (17)	0.32023 (11)	0.28965 (10)	0.0432 (3)	
C6	0.5793 (4)	0.2384 (3)	−0.34382 (18)	0.0925 (7)	
H13A	0.6391	0.3164	−0.3456	0.139*	
H13B	0.4672	0.2312	−0.3475	0.139*	
H13C	0.5901	0.1917	−0.4027	0.139*	
C15	0.9106 (2)	0.42822 (13)	0.31145 (12)	0.0552 (3)	
H18	0.9995	0.4456	0.2790	0.066*	
C19	0.6653 (2)	0.29372 (14)	0.33749 (12)	0.0529 (3)	
H14	0.5888	0.2218	0.3207	0.063*	
C17	0.7585 (3)	0.47953 (16)	0.43073 (12)	0.0646 (4)	
C16	0.8904 (3)	0.51050 (14)	0.38215 (14)	0.0668 (5)	
H17	0.9631	0.5839	0.3963	0.080*	
C18	0.6454 (2)	0.37356 (17)	0.41029 (14)	0.0639 (4)	
H15	0.5581	0.3557	0.4442	0.077*	
O3	1.11026 (12)	0.12734 (10)	0.04622 (11)	0.0629 (3)	
O4	0.72018 (12)	−0.08983 (8)	0.04584 (8)	0.0502 (2)	
C20	0.75917 (14)	−0.15375 (10)	−0.01671 (9)	0.0371 (2)	
C12	1.2107 (4)	−0.0756 (3)	0.52625 (19)	0.0975 (8)	
H20A	1.3259	−0.0563	0.5335	0.146*	
H20B	1.1647	−0.1561	0.5256	0.146*	
H20C	1.1861	−0.0360	0.5843	0.146*	
O7	0.6629 (3)	0.5287 (2)	0.57701 (14)	0.1149 (7)	
N4	0.7411 (3)	0.56459 (19)	0.50990 (14)	0.0906 (6)	
O8	0.8087 (4)	0.66309 (18)	0.50708 (18)	0.1545 (11)	
C21	0.63841 (13)	−0.26916 (9)	−0.05861 (9)	0.0351 (2)	
C22	0.48941 (15)	−0.30059 (11)	−0.02844 (11)	0.0436 (3)	
H25	0.4642	−0.2502	0.0173	0.052*	
C26	0.67611 (16)	−0.34382 (11)	−0.12700 (11)	0.0464 (3)	
H26	0.7759	−0.3226	−0.1472	0.056*	
C24	0.41926 (16)	−0.47908 (11)	−0.13350 (11)	0.0450 (3)	
C23	0.37778 (16)	−0.40726 (12)	−0.06645 (12)	0.0488 (3)	
H29	0.2776	−0.4292	−0.0468	0.059*	
C25	0.56483 (18)	−0.45045 (12)	−0.16547 (12)	0.0519 (3)	
H27	0.5885	−0.5011	−0.2118	0.062*	
N3	0.30228 (18)	−0.59308 (11)	−0.17352 (12)	0.0619 (4)	
O6	0.17528 (17)	−0.61927 (12)	−0.14453 (14)	0.0885 (5)	
O5	0.3380 (2)	−0.65513 (12)	−0.23506 (16)	0.1027 (6)	

### Atomic displacement parameters (Å^2^)

**Table:**

	*U*^11^	*U*^22^	*U*^33^	*U*^12^	*U*^13^	*U*^23^
Mn1	0.03514 (10)	0.03326 (10)	0.04133 (11)	0.00359 (7)	0.00826 (7)	−0.00303 (7)
N1	0.0530 (6)	0.0418 (6)	0.0483 (6)	0.0099 (5)	0.0026 (5)	−0.0001 (5)
N2	0.0387 (5)	0.0488 (6)	0.0485 (6)	0.0070 (5)	0.0100 (4)	0.0052 (5)
C1	0.0444 (7)	0.0508 (8)	0.0530 (7)	0.0058 (6)	0.0067 (6)	0.0035 (6)
C2	0.0559 (9)	0.0651 (10)	0.0518 (8)	0.0134 (7)	0.0004 (6)	0.0009 (7)
C4	0.0625 (10)	0.0703 (11)	0.0750 (11)	0.0090 (8)	0.0192 (8)	0.0299 (9)
C5	0.0465 (8)	0.0635 (10)	0.0671 (10)	0.0002 (7)	0.0075 (7)	0.0163 (8)
C3	0.0668 (10)	0.0764 (11)	0.0482 (8)	0.0293 (9)	0.0148 (7)	0.0126 (7)
C11	0.0932 (13)	0.0409 (7)	0.0563 (9)	0.0073 (8)	−0.0085 (9)	0.0009 (6)
C7	0.0692 (11)	0.0588 (10)	0.0678 (10)	−0.0029 (8)	−0.0121 (8)	0.0113 (8)
C9	0.0855 (12)	0.0702 (11)	0.0515 (8)	0.0401 (9)	0.0027 (8)	0.0043 (7)
C8	0.0787 (13)	0.0807 (13)	0.0687 (11)	0.0079 (10)	−0.0214 (10)	0.0055 (10)
C10	0.1193 (18)	0.0469 (9)	0.0643 (10)	0.0218 (10)	−0.0049 (11)	0.0090 (8)
O2	0.0551 (6)	0.0512 (6)	0.0608 (6)	0.0120 (5)	0.0226 (5)	−0.0055 (5)
O1	0.0568 (6)	0.0422 (5)	0.0649 (6)	0.0086 (4)	0.0177 (5)	−0.0055 (4)
C13	0.0488 (7)	0.0408 (6)	0.0414 (6)	0.0162 (5)	0.0085 (5)	0.0004 (5)
C14	0.0519 (7)	0.0416 (6)	0.0390 (6)	0.0203 (5)	0.0080 (5)	0.0033 (5)
C6	0.1128 (19)	0.1115 (19)	0.0593 (11)	0.0439 (15)	0.0106 (11)	0.0272 (12)
C15	0.0659 (9)	0.0451 (7)	0.0507 (7)	0.0128 (7)	0.0126 (7)	−0.0009 (6)
C19	0.0585 (8)	0.0535 (8)	0.0534 (7)	0.0251 (7)	0.0162 (6)	0.0064 (6)
C17	0.0943 (13)	0.0664 (10)	0.0448 (7)	0.0498 (10)	0.0064 (8)	−0.0035 (7)
C16	0.0943 (13)	0.0436 (8)	0.0567 (9)	0.0219 (8)	0.0038 (9)	−0.0056 (6)
C18	0.0770 (11)	0.0768 (11)	0.0540 (8)	0.0444 (10)	0.0213 (8)	0.0061 (8)
O3	0.0379 (5)	0.0517 (6)	0.0875 (8)	−0.0050 (4)	0.0227 (5)	−0.0058 (5)
O4	0.0497 (5)	0.0379 (5)	0.0530 (5)	0.0022 (4)	0.0110 (4)	−0.0086 (4)
C20	0.0335 (5)	0.0327 (5)	0.0383 (5)	0.0035 (4)	0.0020 (4)	0.0020 (4)
C12	0.123 (2)	0.1125 (19)	0.0656 (12)	0.0615 (17)	−0.0041 (13)	0.0184 (12)
O7	0.1355 (16)	0.1494 (18)	0.0750 (10)	0.0728 (14)	0.0291 (10)	−0.0241 (11)
N4	0.1326 (17)	0.0931 (14)	0.0602 (9)	0.0709 (13)	0.0042 (10)	−0.0155 (9)
O8	0.302 (4)	0.0794 (12)	0.1036 (15)	0.0949 (18)	0.0474 (18)	−0.0120 (10)
C21	0.0308 (5)	0.0315 (5)	0.0377 (5)	0.0042 (4)	0.0042 (4)	0.0016 (4)
C22	0.0354 (5)	0.0406 (6)	0.0504 (7)	0.0056 (5)	0.0109 (5)	0.0000 (5)
C26	0.0397 (6)	0.0387 (6)	0.0547 (7)	0.0031 (5)	0.0150 (5)	−0.0045 (5)
C24	0.0412 (6)	0.0326 (5)	0.0486 (6)	−0.0010 (5)	−0.0018 (5)	0.0048 (5)
C23	0.0336 (6)	0.0460 (7)	0.0588 (8)	0.0003 (5)	0.0096 (5)	0.0070 (6)
C25	0.0540 (8)	0.0366 (6)	0.0567 (8)	0.0053 (5)	0.0114 (6)	−0.0079 (5)
N3	0.0581 (8)	0.0389 (6)	0.0681 (8)	−0.0058 (5)	−0.0046 (6)	0.0061 (6)
O6	0.0597 (7)	0.0637 (8)	0.1117 (12)	−0.0219 (6)	0.0109 (7)	0.0083 (8)
O5	0.1001 (12)	0.0485 (7)	0.1288 (14)	−0.0105 (7)	0.0213 (10)	−0.0325 (8)

### Geometric parameters (Å, º)

**Table:**

Mn1—O3	2.1122 (10)	C6—H13A	0.9600
Mn1—O4	2.1328 (9)	C6—H13B	0.9600
Mn1—N2	2.2621 (12)	C6—H13C	0.9600
Mn1—O2	2.2672 (11)	C15—C16	1.394 (2)
Mn1—N1	2.2746 (13)	C15—H18	0.9300
Mn1—O1	2.3285 (11)	C19—C18	1.388 (2)
Mn1—C13	2.6155 (13)	C19—H14	0.9300
Mn1—Mn1^i^	4.1324 (4)	C17—C18	1.371 (3)
N1—C11	1.323 (2)	C17—C16	1.385 (3)
N1—C7	1.333 (2)	C17—N4	1.478 (2)
N2—C5	1.337 (2)	C16—H17	0.9300
N2—C1	1.3398 (18)	C18—H15	0.9300
C1—C2	1.372 (2)	O3—C20^i^	1.2358 (15)
C1—H1	0.9300	O4—C20	1.2523 (16)
C2—C3	1.381 (3)	C20—O3^i^	1.2358 (15)
C2—H2	0.9300	C20—C21	1.5065 (15)
C4—C5	1.372 (3)	C12—H20A	0.9600
C4—C3	1.389 (3)	C12—H20B	0.9600
C4—H4	0.9300	C12—H20C	0.9600
C5—H5	0.9300	O7—N4	1.230 (3)
C3—C6	1.510 (3)	N4—O8	1.198 (3)
C11—C10	1.378 (3)	C21—C26	1.3852 (17)
C11—H10	0.9300	C21—C22	1.3889 (16)
C7—C8	1.377 (3)	C22—C23	1.3909 (18)
C7—H6	0.9300	C22—H25	0.9300
C9—C10	1.373 (3)	C26—C25	1.3905 (17)
C9—C8	1.378 (3)	C26—H26	0.9300
C9—C12	1.507 (3)	C24—C23	1.369 (2)
C8—H7	0.9300	C24—C25	1.372 (2)
C10—H9	0.9300	C24—N3	1.4779 (16)
O2—C13	1.2495 (17)	C23—H29	0.9300
O1—C13	1.2460 (17)	C25—H27	0.9300
C13—C14	1.5115 (18)	N3—O6	1.204 (2)
C14—C19	1.383 (2)	N3—O5	1.212 (2)
C14—C15	1.388 (2)		
			
O3—Mn1—O4	120.82 (4)	O1—C13—O2	122.88 (12)
O3—Mn1—N2	89.91 (5)	O1—C13—C14	119.14 (12)
O4—Mn1—N2	89.04 (4)	O2—C13—C14	117.98 (12)
O3—Mn1—O2	88.38 (4)	O1—C13—Mn1	62.88 (7)
O4—Mn1—O2	150.77 (4)	O2—C13—Mn1	60.06 (7)
N2—Mn1—O2	89.75 (5)	C14—C13—Mn1	176.03 (10)
O3—Mn1—N1	88.27 (5)	C19—C14—C15	120.42 (13)
O4—Mn1—N1	90.22 (4)	C19—C14—C13	120.16 (13)
N2—Mn1—N1	177.33 (4)	C15—C14—C13	119.42 (13)
O2—Mn1—N1	92.15 (4)	C3—C6—H13A	109.5
O3—Mn1—O1	144.94 (4)	C3—C6—H13B	109.5
O4—Mn1—O1	94.05 (4)	H13A—C6—H13B	109.5
N2—Mn1—O1	94.59 (4)	C3—C6—H13C	109.5
O2—Mn1—O1	56.95 (4)	H13A—C6—H13C	109.5
N1—Mn1—O1	88.02 (5)	H13B—C6—H13C	109.5
O3—Mn1—C13	116.67 (4)	C14—C15—C16	119.86 (16)
O4—Mn1—C13	122.47 (4)	C14—C15—H18	120.1
N2—Mn1—C13	93.26 (4)	C16—C15—H18	120.1
O2—Mn1—C13	28.53 (4)	C14—C19—C18	120.55 (16)
N1—Mn1—C13	89.30 (4)	C14—C19—H14	119.7
O1—Mn1—C13	28.44 (4)	C18—C19—H14	119.7
O3—Mn1—Mn1^i^	44.99 (3)	C18—C17—C16	123.28 (14)
O4—Mn1—Mn1^i^	75.87 (3)	C18—C17—N4	118.56 (19)
N2—Mn1—Mn1^i^	87.25 (3)	C16—C17—N4	118.15 (19)
O2—Mn1—Mn1^i^	133.23 (3)	C17—C16—C15	117.91 (16)
N1—Mn1—Mn1^i^	90.09 (3)	C17—C16—H17	121.0
O1—Mn1—Mn1^i^	169.73 (3)	C15—C16—H17	121.0
C13—Mn1—Mn1^i^	161.66 (3)	C17—C18—C19	117.93 (17)
C11—N1—C7	116.50 (15)	C17—C18—H15	121.0
C11—N1—Mn1	122.21 (11)	C19—C18—H15	121.0
C7—N1—Mn1	121.12 (11)	C20^i^—O3—Mn1	178.20 (10)
C5—N2—C1	116.88 (14)	C20—O4—Mn1	116.68 (8)
C5—N2—Mn1	118.98 (10)	O3^i^—C20—O4	123.88 (11)
C1—N2—Mn1	124.14 (10)	O3^i^—C20—C21	118.52 (11)
N2—C1—C2	123.16 (15)	O4—C20—C21	117.61 (10)
N2—C1—H1	118.4	C9—C12—H20A	109.5
C2—C1—H1	118.4	C9—C12—H20B	109.5
C1—C2—C3	120.12 (15)	H20A—C12—H20B	109.5
C1—C2—H2	119.9	C9—C12—H20C	109.5
C3—C2—H2	119.9	H20A—C12—H20C	109.5
C5—C4—C3	119.99 (16)	H20B—C12—H20C	109.5
C5—C4—H4	120.0	O8—N4—O7	124.4 (2)
C3—C4—H4	120.0	O8—N4—C17	118.2 (2)
N2—C5—C4	123.14 (16)	O7—N4—C17	117.4 (2)
N2—C5—H5	118.4	C26—C21—C22	120.08 (11)
C4—C5—H5	118.4	C26—C21—C20	120.01 (10)
C2—C3—C4	116.67 (15)	C22—C21—C20	119.91 (11)
C2—C3—C6	122.02 (18)	C21—C22—C23	120.16 (12)
C4—C3—C6	121.31 (19)	C21—C22—H25	119.9
N1—C11—C10	123.56 (17)	C23—C22—H25	119.9
N1—C11—H10	118.2	C21—C26—C25	120.02 (12)
C10—C11—H10	118.2	C21—C26—H26	120.0
N1—C7—C8	123.10 (17)	C25—C26—H26	120.0
N1—C7—H6	118.5	C23—C24—C25	123.12 (11)
C8—C7—H6	118.5	C23—C24—N3	118.70 (13)
C10—C9—C8	116.18 (16)	C25—C24—N3	118.17 (14)
C10—C9—C12	121.6 (2)	C24—C23—C22	118.22 (12)
C8—C9—C12	122.2 (2)	C24—C23—H29	120.9
C7—C8—C9	120.40 (18)	C22—C23—H29	120.9
C7—C8—H7	119.8	C24—C25—C26	118.39 (13)
C9—C8—H7	119.8	C24—C25—H27	120.8
C9—C10—C11	120.27 (18)	C26—C25—H27	120.8
C9—C10—H9	119.9	O6—N3—O5	123.18 (15)
C11—C10—H9	119.9	O6—N3—C24	118.46 (16)
C13—O2—Mn1	91.41 (8)	O5—N3—C24	118.36 (15)
C13—O1—Mn1	88.68 (8)		

Symmetry code: (i) −*x*+2, −*y*, −*z*.

### Hydrogen-bond geometry (Å, º)

Cg is the centroid of the N2/C1–C5 ring.

**Table:**

*D*—H···*A*	*D*—H	H···*A*	*D*···*A*	*D*—H···*A*
C1—H1···O4	0.93	2.61	3.172 (2)	119
C2—H2···O1^ii^	0.93	2.65	3.278 (2)	125
C11—H10···O4	0.93	2.55	3.161 (2)	124
C22—H25···*Cg*^ii^	0.93	2.80	3.6844 (16)	160

Symmetry code: (ii) −*x*+1, −*y*, −*z*.
